# Fine-Needle Aspiration Cytology in the Diagnosis of Salivary Gland Tumors Before the Milan System: A Ten-Year Experience From a Tertiary Care Center in Greece

**DOI:** 10.7759/cureus.42737

**Published:** 2023-07-31

**Authors:** Constantinos Mourouzis, Ourania Schoinohoriti, Dimitris Mastagkas, George Rallis

**Affiliations:** 1 Department of Oral and Maxillofacial Surgery, KAT Attica General Hospital, Athens, GRC; 2 Department of Oral and Maxillofacial Surgery, Dental School, University of Athens, Athens, GRC; 3 Department of Oral and Maxillofacial Surgery, 401 Military Hospital of Athens, Athens, GRC

**Keywords:** accuracy, diagnostic value, benign and malignant tumors, salivary glands, fine needle aspiration cytology

## Abstract

Objective

The objective of this study was to determine the diagnostic value of fine-needle aspiration cytology (FNAC) for salivary gland tumors.

Methodology

A retrospective file analysis of patients with salivary gland pathology, attending the Department of Oral and Maxillofacial Surgery of a tertiary care center in Athens, Greece, over a 10-year-long period, was conducted. Sensitivity, specificity, accuracy, positive prognostic value (PPV), and negative prognostic value (NPV) of FNAC for benign and malignant tumors separately were assessed and compared with histology.

Results

A total of 82 patients (46 male and 36 female) with salivary gland tumors, submitted to both FNAC and histology, were included. The mean age was 55 years. A total of 73 tumors were histologically diagnosed as benign and nine as malignant. FNAC identified 62 benign and seven malignant tumors but was inconclusive in 13 cases. The most common diagnosis of both histology and FNAC was pleomorphic adenoma. FNAC sensitivity, specificity, accuracy, PPV, and NPV were 98.3% and 100%, 87.5% and 100%, 97.1% and 100%, 98.3% and 100%, and 87.5% and 100% for benign and malignant tumors, respectively.

Conclusions

FNAC is highly sensitive but moderately specific for the preoperative identification of benign salivary gland tumors. Its use as an initial diagnostic modality is warranted, thanks to its safeness, rapidity, and lack of pain.

## Introduction

Salivary gland tumors are clinically diverse, with an estimated overall annual incidence of approximately 2.5-3 cases per 100,000 in the Western world [1]. Salivary gland malignancies account for approximately 3% to 6% of head and neck tumors, with an overall annual worldwide incidence of 0.4-13.6 cases per 100,000, with 80% affecting the parotid gland [2]. The World Health Organization classifies salivary gland epithelial tumors into 10 benign and 23 malignant entities, while nonepithelial represent only about 2% to 5%.

Fine-needle aspiration cytology (FNAC) is a diagnostic tool, used widely thanks to its safeness, cost-effectiveness, rapidity, repeatability in case of nondiagnostic results, and lack of pain [3]. Performed by experienced operators and correlated with clinical information, it effectively diagnoses the nature of the lesion. Its varying accuracy has been ascribed to many reasons. Nevertheless, fine-needle aspiration (FNA) has been related to complications, such as neoplastic cell seeding, bleeding, inflammatory reaction, and/or nerve impairment [4-6].

Especially in the diagnosis of salivary gland tumors, FNAC is becoming popular, due to the tumors' superficial location and easy accessibility. Given the noncharacteristic clinical and/or radiologic features of these tumors, FNAC is considered superior to combined physical and radiologic examination [5,7,8]. Recently, ultrasound-guided core biopsy has been studied as an alternative for the preoperative evaluation of parotid lesions, but its use as a primary diagnostic tool is restricted by the high incidence of hematoma and facial nerve injury [9].

In 2018, an international group of experts, supported by the American Society of Cytopathology and the International Academy of Cytology, introduced the Milan System for Reporting Salivary Gland Cytopathology (MSRSGC), containing various diagnostic categories (subdivisions of FNAC results), their appurtenant risk of malignancy, and advised management strategy [10].

The objective of the study was to determine the diagnostic value of FNAC for salivary gland tumors among patients, who were attending the Department of Oral and Maxillofacial Surgery of a tertiary care center in Athens, Greece, during a 10-year-long period in the pre-MSRSGC era and compare our findings with those reported in the recent literature.

## Materials and methods

The files of all patients with salivary gland (including parotid, submandibular, and minor oral salivary glands) pathology, diagnosed from 2009 to 2019, were retrieved from the database and analyzed retrospectively. Harvested data included patients’ demographics and history, lesion characteristics (location and size), as well as histological and FNAC diagnoses.

FNAs had been performed by specialized clinicians of our department, without ultrasonography assistance, by using a 22-gauge needle, with a minimum of two needle passes in each case, while an experienced cytopathologist was present to assess the slide adequacy immediately after sample collection.

FNAC findings were classified into the following categories: (1) nondiagnostic or inconclusive, (2) inflammatory/nonneoplastic lesions, (3) benign tumors, and (4) malignancies. Nondiagnostic results and inflammatory/nonneoplastic lesions were not submitted for statistical analysis.

For this study, each tumor was classified as either benign or malignant.

Benign tumors

Benign tumors were classified into four categories: (1) true positive (TP), cases diagnosed as benign tumors both cytologically and histologically; (2) true negative (TN), cases diagnosed as malignancies both cytologically and histologically; (3) false positive (FP), cases initially diagnosed as benign tumors cytologically but not confirmed histologically as benign; and (4) false negative (FN), cases not identified as benign tumors by FNAC, but later diagnosed as benign on histological examination.

Malignant tumors

Malignant tumors were classified into four categories: (1) TP, cases diagnosed as malignant tumors both cytologically and histologically; (2) TN, cases diagnosed as non-malignant tumors both cytologically and histologically; (c) FP, cases histologically diagnosed as benign tumors but misdiagnosed cytologically as malignancies; and (4) FN, cases misdiagnosed cytologically as benign tumors, but later confirmed histologically as malignancies.

Sensitivity, specificity, negative prognostic value (NPV), and positive prognostic value (PPV) of FNAC for benign and malignant tumors of the salivary glands were evaluated separately, as follows:

Sensitivity = TP / TP + FN

Specificity = TN / TN + FP

PPV = TP / TP + FP

NPV = TN / TN + FN

Total diagnostic accuracy = TP + TN / total number of cases

Definite histological typing was achieved following tumor excision/resection for all diagnostic FNAC specimens and through incisional biopsies in some of the inconclusive.

Subsequently, FNAC diagnoses were compared with the corresponding histology; discrepancies between FNAC and histology on the histological type of the tumors were registered and analyzed. Kappa statistics were used to assess the agreement between FNAC and histology in the diagnosis of both benign and malignant tumors.

Our results were compared with those of the most relevant articles in English, with a sample size of over 40 patients, published over the last 15 years, providing values for sensitivity, specificity, diagnostic accuracy, PPV, and NPV at least for salivary gland malignancies.

## Results

A total of 82 salivary gland tumors, submitted to both FNAC and histology, were included. One of these (#31), originally misdiagnosed through FNA as pleomorphic adenoma, was histologically typed as a lymphoepithelial cyst. The age range was 27 to 81 (mean 55) years and the male-to-female ratio was 46:36 (0.78). Patients’ data, tumor characteristics, and type of surgery are summarized in Table 1.

**Table 1:** Summarized patients’ data. F, female; M, male; L, left; R, right; PA, pleomorphic adenoma; WT, Warthin’s tumor; par/tomy, parotidectomy; PSP, partial superficial parotidectomy; SCC, squamous-cell carcinoma; MRND III, Modified Radical Neck Dissection Level III

#	Age	Gender	Location	Surgery	FNAC	Histology
1	56	F	Parotid L	Superficial par/tomy	Adenocarcinoma	Adenocarcinoma
2	73	F	Hard palate R	Excision	PA	PA
3	61	F	Parotid R	PSP	PA	PA
4	75	M	Hard palate L	Incisional biopsy	Mucoepidermoid carcinoma	Mucoepidermoid carcinoma
5	43	M	Submandibular L	SMG excision	inconclusive	Hodgkin’ s
6	52	M	Parotid R	Total par/tomy	Lymphoid lineage cells	WT
7	47	M	Parotid R	Superficial par/tomy	PA	PA
8	47	M	Parotid R	PSP	WT	PA
9	48	M	Parotid L	PSP	WT	WT
10	47	F	Parotid L	PSP	PA	PA
11	54	M	Parotid L	PSP	WT	WT
12	50	M	Parotid L	Superficial par/tomy	PA	PA
13	60	F	Parotid L	Extracapsular dissection	PA	PA
14	48	F	Parotid L	PSP	inconclusive	Mucoepidermoid carcinoma
15	77	M	Parotid L	Subtotal par/tomy	inconclusive	Warthin’s tumor
16	67	F	Hard palate	Excision	PA	PA
17	43	M	Hard palate	Excision	PA	PA
18	71	M	Parotid L	PSP	Inconclusive	WT
19	63	M	Parotid L	PSP	WT	WT
20	56	M	Parotid L	PSP	WT	WT
21	41	F	Parotid L	Subtotal par/tomy	PA	PA
22	66	F	Parotid R	PSP	Inconclusive	WT
23	46	F	Parotid R	PSP	PA	PA
24	35	Μ	Parotid R	PSP	PA	PA
25	59	Μ	Parotid R	PSP	Inconclusive	WT
26	58	M	Parotid L	PSP	WT	WT
27	72	F	Parotid R	PSP	Inconclusive	WT
28	54	F	Soft palate R	Excision	Inconclusive	PA
29	54	F	Parotid L	Total par/tomy	WT	WT
30	61	M	Parotid L	PSP	WT	WT
31	63	F	Parotid L	PSP	PA	Lymphoepithelial cyst
32	45	M	Parotid L	Extracapsular dissection	PA	PA
33	67	F	Parotid R	PSP	WT	WT
34	56	F	Parotid R	Superficial par/tomy	PA	PA
35	49	M	Parotid L	Extracapsular dissection	PA	PA
36	62	M	Parotid R	PSP	WT	WT
37	61	M	Soft palate R	Excision	PA	PA
38	68	F	Parotid R	PSP	PA	PA
39	43	M	Parotid L	PSP	PA	PA
40	51	M	Parotid L	PSP	PA	PA
41	69	F	Parotid L	Superficial par/tomy	PA	PA
42	45	F	Parotid L	PSP	PA	PA
43	59	M	Parotid R	Superficial par/tomy	inconclusive	WT
44	34	M	Parotid L	Extracapsular dissection	WT	Lipoma
45	61	F	Parotid L	PSP	WT	WT
46	64	M	Parotid R	Superficial par/tomy	Adenocarcinoma	Ductal carcinoma
47	27	F	Parotid R	PSP	PA	PA
48	39	F	Parotid L	Superficial par/tomy	PA	PA
49	35	F	Parotid L	PSP	PA	PA
50	61	M	Parotid L	Subtotal par/tomy	Inconclusive	Basal-cell adenoma
51	50	M	Parotid L	PSP	WT	WT
52	67	M	Parotid R	Subtotal par/tomy	PA	PA
53	67	F	Parotid R	Incision biopsy	SCC	SCC
54	64	F	Parotid R	PSP	Inconclusive	WT
55	33	F	Parotid R	Extracapsular dissection	PA	PA
56	37	M	Upper lip R	Extracapsular dissection	PA	PA
57	27	M	Parotid L	Extracapsular dissection	PA	PA
58	69	M	Parotid R	PSP	WT	WT
59	55	F	Parotid L	PSP	WT	WT
60	27	F	Hard palate R	Excision	PA	PA
61	65	M	Parotid L	PSP	WT	WT
62	54	M	Parotid R	PSP	WT	WT
63	70	M	Hard palate R	Incision biopsy	Adenocarcinoma	Pleomorphic adenocarcinoma
64	48	M	Parotid L	PSP	WT	WT
65	27	F	Parotid R	PSP	PA	PA
66	27	M	Parotid L	Total par/tomy	PA	PA
67	55	M	Parotid L	PSP	WT	WT
68	45	M	Parotid L	Superficial par/tomy	Inconclusive	WT
69	81	M	Parotid L	PSP	WT	WT
70	67	F	Parotid R	PSP	PA	PA
71	73	M	Parotid R	Subtotal par/tomy	WT	WT
72	75	F	Parotid R	PSP	Basal-cell adenoma	Basal-cell adenoma
73	64	M	Submandibular R	Excision + MRND III	Adenocarcinoma	Adenocarcinoma
74	23	F	Parotid R	Extracapsular dissection	PA	PA
75	67	M	parotid L	PSP	Inconclusive	Lipoma
76	45	F	Soft palate L	Extracapsular dissection	PA	PA
77	36	M	Parotid R	PSP	PA	PA
78	77	M	Parotid L	PSP	WT	WT
79	66	F	Parotid L	PSP	PA	PA
80	79	M	Parotid L	Subtotal par/tomy	Adenocarcinoma	Adenocarcinoma
81	65	F	Parotid L	PSP	PA	PA
82	67	F	Parotid L	Total par/tomy	PA	PA

Of the 82 included tumors, 70 affected the parotid, 10 were the minor oral (six of the hard palate, three of the soft palate, and one of the upper lip), and two were the submandibular salivary glands (Table 1).

It is worth mentioning that FNAC was nondiagnostic in 11 benign and two malignant tumors; thus, 13 out of the 82 FNAC specimens (17.07%) were inconclusive.

Of the 81 true tumors (excluding case #31), 72 were benign and nine were malignant. Among the benign tumors, pleomorphic adenoma was the most common (39/72, 54.16%), followed by Warthin’s tumor (29/72, 40.27%); the remaining diagnoses were lipoma (2/72, 0.27%) and basal-cell adenoma (2/72, 0.27%). Among malignancies, adenocarcinoma was the most common (3/9, 33.33%), followed by mucoepidermoid carcinoma (2/9, 22.22%); the remaining diagnoses were Hodgkin’s lymphoma, ductal carcinoma, squamous-cell carcinoma, and pleomorphic adenocarcinoma (1/9 for each, 11.1%; Table 2).

**Table 2:** Evaluation of the FNAC diagnostic value, including sensitivity, specificity, PPV, NPV, and diagnostic accuracy, as well as Kappa statistics for benign and malignant tumors. FNAC, fine-needle aspiration cytology; NPV, negative prognostic value; PPV, positive prognostic value

Variable	Benign lesions (95% CI )	Malignant lesions (95% CI )
Sensitivity (%)	98.36 (0.91-0.99)	100 (0.59-1)
Specificity (%)	87.5 (0.47-0.99)	100 (0.94-1)
PPV (%)	98.36 (0.91-0.99)	100 (0.59-1)
NPV (%)	87.5 (0.47-0.99)	100 (0.94-1)
Kappa	0.85 (0.62-1.09)	1
Prevalence (%)	88.4 (0.78-0.94)	10.14 (0.04-0.19)
Diagnostic accuracy (%)	97.1 (0.89-0.99)	100 (0.94-1)

To determine FNAC efficacy in histological typing, the cytological diagnosis of each tumor was compared with histology (Table 2). In most cases, FNAC results were consistent with histology. Histological typing was accurate in 93.54% (58/62) of the benign tumors, except for one FP (#31, i.e., a lymphoepithelial cyst with FNAC diagnosis *pleomorphic adenoma*) and one FN (#6, i.e., a Warthin’s tumor with FNAC diagnosis *lymphoid lineage cells*). Moreover, FNAC correctly detected seven out of the nine malignancies (71.43% accuracy) but was inconclusive in two cases, diagnosed histologically as Hodgkin’s lymphoma and mucoepidermoid carcinoma; thus, there were no FP or FN cases among malignancies. K statistics for the agreement degree between FNAC and histology are presented in Table 3.

**Table 3:** Correlation of FNAC and histology diagnoses of the benign and malignant tumors of the study. FNAC, fine-needle aspiration cytology

Benign tumors	FNAC diagnosis	Histopathology diagnosis	Total
	Pleomorphic adenoma	Pleomorphic adenoma	37
	inconclusive	Pleomorphic adenoma	1
	Warthin’s tumor	Warthin’s tumor	20
	Pleomorphic adenoma	Benign lymphoepithelial cyst	1
	Inconclusive	Warthin’s tumor	8
	Lymphoid lineage cells	Warthin’s tumor	1
	Warthin’s tumor	Pleomorphic adenoma	1
	Inconclusive	Lipoma	1
	Warthin’s tumor	Lipoma	1
	Basal-cell adenoma	Basal-cell adenoma	1
	Inconclusive	Basal-cell adenoma	1
	Total		73
Malignant tumors	FNAC diagnosis	Histology diagnosis	Total
	Adenocarcinoma	Adenocarcinoma	3
	Mucoepidermoid carcinoma	Adenocarcinoma	1
	Adenocarcinoma	Ductal carcinoma	1
	Mucoepidermoid carcinoma	Mucoepidermoid carcinoma	1
	Adenocarcinoma	Pleomorphic adenocarcinoma	1
	Inconclusive	Hodgkin’s lymphoma	1
	inconclusive	Mucoepidermoid carcinoma	1
	Total		9

The diagnostic value of FNAC for identifying salivary gland tumors, including sensitivity, specificity, PPV, NPV, and accuracy for both benign and malignant tumors are presented in Table 3.

## Discussion

FNAC has been gaining acceptance as an adjunct for preoperative diagnosis of salivary gland pathology, obviating surgery in approximately 33% of the patients, and providing useful information about the extent and time of indicated surgery [10-12]. It speeds up the diagnostic process by providing a diagnosis within 24 hours, whereas histopathology may take five to seven days, thus reducing the morbidity and mortality associated with malignancies [13]. FNAC is a safe, cost-effective, and minimally invasive procedure that causes little patient discomfort. However, it carries a low risk of malignant cell dissemination [5,8]. Due to the morphological complexity of salivary gland tumors, the relatively small FNAC specimen may not be representative, with varying proportions (2%-10%) of inadequate smears [14,15]. Ultrasonography-assisted FNAC has been recommended to reduce sampling errors [16,17]. Variables such as the operator’s experience, applied technique, promptness of sample evaluation, and the cytopathologist’s diagnostic skills influence FNAC accuracy [3,14,16,17].

Especially for the parotid gland, FNAC is included in the diagnostic workup to (a) rule out inflammation, (b) identify systemic disease (reticuloendothelial tumors), (c) exclude direct gland invasion or metastases in patients with oncologic history, (d) evaluate unresectable tumors in poor surgical candidates, and (e) assess patients with low risk of neoplasms, such as children. Preoperative FNAC in parotid malignancies allows staging, determines the surgical plan (resection margins, need for neck dissection, and urgency degree), and assesses the risk of postoperative facial palsy [10,15]. Histology following incisional biopsy is still considered the gold standard to evaluate malignant potential, although potentially leads to fistula or sialocele formation, tumor implantation, or facial nerve damage [5,6].

FNA-induced tissue alterations, such as necrosis, hemorrhage, cellular atypia (squamous metaplasia), cellular proliferation, displacement through the tumor capsule, and/or architecture distortion (stromal hyalinization) that confound subsequent histological diagnosis, have been reported [14]. However, FNAC benefits outweigh the risk of distortion beyond histological recognition, thus not downgrading its diagnostic value.

The value of FNAC has been questioned due to its low sensitivity and high rate of FN results. Its sensitivity is generally lower and more variable (52%-98%) than specificity (56%-100%), while diagnostic accuracy ranges from 78% to 98% [1,5,11,16,18-28]. A review of the recent literature on the diagnostic value of FNAC for salivary gland pathology is summarized in Table 4 [1-5,8,11-13,15-17,19-28].

**Table 4:** Recent literature review on the diagnostic value of FNAC for determining salivary gland malignancy. PPV, positive prognostic value; NPV, negative prognostic value; FNAC, fine-needle aspiration cytology

Study	Year	Total FNAC cases	Sensitivity (%)	Specificity (%)	Accuracy (%)	PPV (%)	NPV (%)
Jafari et al. [19]	2009	110	67	96	-	80	-
Ashraf et al. [20]	2009	100	77.77	98.78	-	93.33	95.29
Ali et al. [4]	2011	129	84	98	94	93	95
Hartimath et al. [5]	2011	51	90.9	96.6	95	90	96.6
Piccioni et al. [3]	2011	176	81	99	97	93	98
Fakhry et al. [15]	2012	202	80	89.5	86.5	73	79.6
Javadi et al. [21]	2012	65	57.9	97.8	86	91.7	84.9
Nguansangiam et al. [22]	2012	133	81.3	99.1	97	92.9	97.5
Singh Nanda et al. [23]	2012	56	84.61	86.48	-	68.75	94.11
Jain et al. [8]	2013	80	92.8	93.9	-	81.2	98.4
Jeong et al. [24]	2013	158	39.1	99.3	77.8	90	90.5
Mallon et al. [11]	2013		52	98	92	78	93
Feinstein et al. [12]	2016	272	75	95.1	-	84.9	91.2
Ghantous et al. [25]	2016	79	90	98	88	90	98
Gudmundsson et al. [1]	2016	114	73	97	95	73	97
Mohammed Nur and Murphy [16]	2016	262	80.7	98.2	-	-	-
Shetty and Geethamani [26]	2016	114	94.4	100	97.6	-	-
Marzouki et al. [2]	2017	42	50	100	92.1	100	91.4
Ramírez-Pérez et al. [17]	2017	144	60	97.5	-	83.3	92
Eytan et al. [27]	2018	451	82.4	90.4	87.8	80.8	91.3
Shalley et al. [13]	2018	65	89.5	100	85	-	-
Yildiz et al. [28]	2021	208	59.09	97.85	93.75	76.47	95.29

This remarkable discrepancy was partly ascribed to FNAC’s dependency on the operator’s and cytopathologist’s skills and experience, the preparation and fixation of slides, and the defined inadequacy threshold [4,16,17,24,25,29]. It was also related to the noncomparability of data, harvested from different patient populations, by using different methodologies [11,17,22,28-30]. Some authors analyzed together major and minor salivary glands, while others focused only on the parotid gland [22,23,29,30]. Moreover, sensitivity, specificity, and diagnostic accuracy were calculated separately either for malignant and benign lesions or for each single histological subtype [11,23,24,29]. In some studies, FNAC was performed routinely, while in others, only in cases suspected of malignancy, reducing the prevalence of malignancy and affecting FNAC’s diagnostic accuracy [3,17,29].

Our results showed 98.36% and 100% sensitivity, 87.5% and 100% specificity for benign and malignant tumors, respectively; PPV amounted to 98.36% and 100%, NPV to 87.5% and 100%, and diagnostic accuracy to 97.1% and 100%. The relatively high PPV (lack of FPs) is attributed to the relatively small sample size and the low prevalence of malignancies (10.14%); larger studies are more likely to encounter FPs, thus reducing both PPV and specificity.

According to the Cytopathology Resource Committee of the College of American Pathologists, participating laboratories were inadequate to provide a specific diagnosis, although cytologically differentiating benign from malignant tumors (<50% agreement between FNAC and histology) [18]. Tumor type and grade reportedly affect the diagnostic accuracy of FNAC, with specific tumors contributing disproportionately to diagnostic errors; accurate diagnosis may be achieved in high-grade carcinomas, while negative results are mostly encountered in low-grade carcinomas [14,18]. However, precise preoperative typing is not particularly relevant in malignancy, as accurate grading is the most substantial parameter for subsequent surgical planning (radicality of surgery and need for neck dissection) [14,29].

The failure of FNAC to identify specific salivary gland tumors may be ascribed to their morphological complexity (various growth patterns and diverse cell types) and the limited FNA sample that may not be representative of the complete tumor morphologic spectrum [14,15,21,24]. FNAC demonstrates inadequate tumor invasiveness, failing to diagnose carcinomas such as adenoid cystic carcinoma that are highly infiltrative, although appearing bland and nonthreatening cytologically [14]. FNAC also fails to evaluate malignancy signs, such as capsular infiltration, and perineural and perivascular invasion [30].

The pitfalls of FNAC reportedly lie in the diagnosis of pleomorphic and basal-cell adenomas, as well as carcinomas and lymphomas [1,7,13,26]. In pleomorphic adenoma, a few atypical cells may be present, while monomorphic adenoma can be confused with adenoid cystic carcinoma due to the presence of similar stromal components [13]. FNAC accuracy for the diagnosis of largely cystic tumors (Warthin’s tumor, mucoepidermoid carcinoma, and adenoid cystic carcinoma) is reportedly small, due to the inadequate architectural information provided through FNAC [6,7,14,23]. Mucoepidermoid carcinoma is particularly challenging to diagnose cytologically, as FN diagnoses may occur due to fluid dilution or bland-looking intermediate cells, which can be misinterpreted as benign.

The limitations of FNAC in differentiating reactive lymphoid hyperplasia from lymphoma are well-documented. Lymphocyte-predominant lesions significantly contribute to both FN and FP cytological results. Moreover, FNAC fails to distinguish between reactive lymphoid hyperplasia and granulomas originating from either the salivary glands or the intra-/peri-salivary lymph nodes in the parotid and submandibular region [6].

FNAC histological typing was accurate in 93.54% (58/62) of the benign and 71.43% (5/7) of the malignant tumors, with kappa values 0.85 (95% confidence interval [CI] 0.62-1.09) and 1, respectively. Both benign tumors with disagreeing FNAC and histology were Warthin’s tumors. Malignant tumors with divergent cytological and histological diagnoses were ductal carcinoma (#46) and pleomorphic adenocarcinoma (#63).

The rate of nondiagnostic (inconclusive) FNAC among our patients was 17.07% (13/82). These were excluded from statistical analysis to minimize the risk of overrating FN results. Although consistent with the reported results in the literature (3%-34%) [6,8,9,14], this rate was partly ascribed to the lack of ultrasound guidance, resulting in low diagnostic yield for deeply located parotid tumors or cystic tumors [14,17], such as Warthin’s tumors (8/13 inconclusive cases). However, even in studies with ultrasound-assisted FNA, nondiagnostic results were attributed to complex tissue architecture [3] or inadequate smear cellularity and improper sampling [12]. Moreover, failure to obtain representative specimens may be due to needle positioning outside the tumor or into a central necrosis or hemorrhage area [24]. In our study, all inconclusive FNAC specimens were uninterpretable due to inadequate smears, possibly owing to improper sampling or limited smear cellularity, common in cystic tumors (Warthin’s and mucoepidermoid carcinomas); thus, they were excluded from further analysis to avoid bias of the estimated FNAC diagnostic value. However, one should bear in mind that according to the MSRSGC, nondiagnostic FNAC cases present a 25% risk of malignancy [10].

The retrospective nature of this study is a considerable limitation for the following reasons: (1) some patients' files may have inadequate data, and access to a more comprehensive database would improve patient care and facilitate future studies aiming to understand salivary gland pathology and enhance the accuracy of FNAC; and (2) inability to overcome the invalidity of at least some of our inconclusive FNAC results by repeating the FNA procedure.

## Conclusions

Based on our findings FNAC is highly sensitive and moderately specific for the diagnosis of benign salivary gland tumors, with high specificity for salivary gland malignancies, establishing the need for surgery but not ensuring histological typing.

FNAC is a relatively safe, rapid, and noninvasive method, when compared with an incisional biopsy but plays a limited role in determining the radicality of surgery (spare or sacrifice the facial nerve). Further investigation is required to elucidate factors, defining the diagnostic accuracy of FNAC, that is optimized when combined with clinical and radiologic evaluation.
